# Effects of selenylation on Chinese yam polysaccharides: Structure, antioxidant, and digestive properties

**DOI:** 10.1016/j.fochx.2025.102435

**Published:** 2025-04-04

**Authors:** Weiling Liu, Yujun Jiang, Jia Shi

**Affiliations:** a Department of Food Science, Key Laboratory of Dairy Science, Ministry of Education, Northeast Agricultural University, Harbin 150030, PR China; b Key Laboratory of Infant Formula Food, State Administration for Market Regulation, Harbin 150030, PR China

**Keywords:** Yam polysaccharides, Selenylation, Functional properties, *In vitro* digestion

## Abstract

Natural polysaccharides have unsatisfactory properties in production and processing due to structural limitations. Recent studies have shown that chemical modifications can improve the physicochemical and functional properties of plant polysaccharides. Herein, the effect of selenylation on the structure, functional properties, and *in vitro* digestion characteristics of yam polysaccharide (YP) was investigated. Selenylated products with different selenium contents (YP-LSe and YP-HSe) were prepared by controlling the addition of sodium selenite, and all samples were identified as acidic polysaccharides. Selenylation induced alterations in the chemical composition of YP. FT-IR spectral analysis revealed that YP-LSe and YP-HSe exhibited characteristic vibrational absorption peaks associated with selenium-containing groups. Microstructure analysis showed that YP-LSe and YP-HSe presented stacked leaf-like structures with sphere attachments. Moreover, selenylation significantly enhanced the emulsion capacity, foaming capacity, and antioxidant capacity of YP. In the simulated digestion process, YP-LSe and YP-HSe exhibited greater resilience against the gastrointestinal environment than YP. This study provides a theoretical basis for the development and utilization of selenylation of YP in the field of functional foods.

## Highlights

• Selenylation modification altered the chemical composition of YP.
• Selenylated YP exhibited stacked leaf-like structure with sphere attachments.
• Selenylation modification improved functional and antioxidant properties of YP.
• Selenylated YP exhibited stronger gastrointestinal tolerance than YP.

## 1 Introduction

Yam (*Dioscorea opposita* Thunb*.*), the world's fourth largest tropical root crop following cassava and sweet potato, is a traditional crop with a dual origin in both medicine and food (Zhang et al., 2024). The tubers of yam are rich in bioactive compounds, including polysaccharides, allantoin, flavonoids, alkaloids, and diosgenin. Among these, yam polysaccharides (YP) are the primary active constituents, predominantly composed of mannose, xylose, arabinose, glucose, and galactose (Liu et al., 2022). Some studies showed that YP exhibited various biological activities, such as hypoglycemic effects, promoting growth, antioxidation, and modulating immune responses (Zhang et al., 2024). However, the properties of YP are unsatisfactory in production and processing due to structural limitations. Therefore, the chemical modification of polysaccharides has been extensively studied. Natural polysaccharides can be chemically modified to introduce new functional groups, alter molecular weight, and modify their structure, thereby enhancing their activity (Guan & Zhao, 2022). Current methods of polysaccharide modification include sulphation, carboxymethylation, acetylation, phosphorylation, and selenylation (Wang, Zheng, et al., 2024). Phosphorylation and acetylation present considerable challenges in controlling reactions. Carboxymethylation often requires toxic organic reagents, while sulfation is prone to inducing carbonization. Selenylation has several advantages, such as straightforward reaction conditions, a precisely controllable reaction process, high efficiency, and improved safety (Chen, Shen, et al., 2024). It is worth noting that selenylation can produce modified polysaccharides with both selenium and polysaccharide activities, which is a promising approach to enhance the functional properties of polysaccharides and broaden their application prospects.

Selenium is an essential trace element with biological effects that are conducive to growth and development. Selenium is a component of the enzyme that activates thyroid hormones, playing roles in regulating the thyroid gland and boosting immunity (Winther et al., 2020). Selenium is involved in the synthesis of glutathione peroxidase (GSH-Px) and superoxide dismutase (SOD) to protect the body from oxidative damage caused by radicals (Cheng et al., 2018). The chemical forms of selenium can be categorized into inorganic selenium, which has low bioavailability and potential toxicity, and organic selenium, which is the predominant form in the body. Organic selenium includes selenium amino acids, selenoproteins, and selenium-containing polysaccharides (Huang et al., 2020). Polysaccharides are abundant in hydroxyl groups, which can participate in nucleophilic substitution reactions with electrophilic selenium species. This interaction facilitates the alteration of selenium's valence state, thereby transforming inorganic selenium into organic selenium. Selenylated polysaccharides exhibited bioactivity characteristics of both selenium and polysaccharides that can also be used as potential selenium-sourced dietary supplements. For instance, the introduction of selenium provided the thermal stability and antioxidant properties of *Morchella sextelata* polysaccharide (Deng et al., 2024). Selenium biofortification of *Pleurotus eryngii* polysaccharides had the potential to prevent food-derived heavy metal hazards (Wang, Ji, et al., 2024). However, previous studies have mostly focused on the effects of selenylation on the biological activity of polysaccharides, and there is relatively little research on the structural and functional properties of polysaccharides with different degrees of selenylation. The specific effects of the selenylation on the molecular structure, functional attributes, and *in vitro* digestibility of YP remain to be fully elucidated, warranting further comprehensive investigation.

The aim of this study was to explore the effects of covalent selenylation on the intrinsic structural and functional properties of YP. Using the HNO_3_-Na_2_SeO_3_ system, YP was combined with sodium selenite and differentiated based on the amount of sodium selenite added. Two different degrees of selenylation of YP were synthesized, named YP-LSe and YP-HSe, respectively. The effects of selenylation on the chemical composition and microstructure of YP were analyzed. The possible relationship between the degree of selenylation and the functional properties of polysaccharides was investigated by the study of emulsification, foaming, antioxidant activity, and *in vitro* digestibility. The findings of this research are expected to provide new insights into the application of YP in the field of functional foods.

## 2 Materials and methods

### 2.1 Materials

The fresh yams at the commercially mature stage were purchased from the local market in Harbin. The α-amylase was purchased from Solarbio Biotechnology Co., Ltd. (Beijing, China). Alkaline protease was purchased from Beijing Aoboxing Biotechnology Co., Ltd. (Beijing, China). Sodium selenite was purchased from Sinopharm Chemical Reagent Co., Ltd. (Shanghai, China). Pepsin was purchased from Dalian Meilun Biotechnology Co., Ltd. (Dalian, Liaoning, China). Trypsin and porcine bile salts were purchased from Shanghai Yuanye Biotechnology Co., Ltd. (Shanghai, China).

### 2.2 Extraction and selenylation of YP

**Fig. 1:**
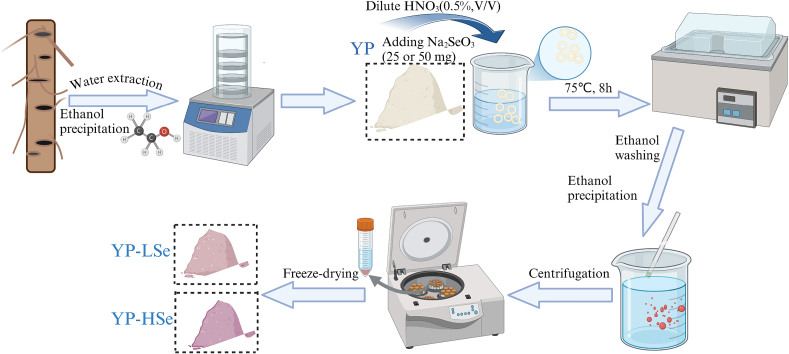
Preparation flow chart of YP-LSe and YP-HSe.

YP was extracted using methods from previous research (Guan & Zhao, 2022). The preparation process of samples was performed according to Fig. 1. Briefly, deionized water was added to the peeled and homogenized yam at a ratio of 10:1 (*w*/w). α-amylase (20 U/mL) was added to the mixture and then heated at 85 °C for 4 h. The mixture was centrifuged at 8000 r/min for 10 min, and the supernatant was treated with alkaline proteinase (100 U/mL) at 55 °C for 6 h. Three times the volume of anhydrous ethanol was added to the concentrated system and maintained for 24 h at 4 °C. The precipitate was washed with anhydrous ethanol and lyophilized to obtain YP.

The recommended dietary intake of selenium for adults is 55 μg/day and the upper intake limit is 400 μg/day (Le et al., 2024). To ensure the maintenance of safe and effecti*v*e selenium levels, the dosage proposed by Yu et al. (2022) was referenced and appropriately adjusted. 0.5 g of YP was dissolved in 80 mL of HNO_3_ (0.5 %, *v*/v). 25 mg and 50 mg of Na_2_SeO_3_ were added to the solution, respectively, and the reaction was carried out for 8 h at 75 °C. Three times the volume of anhydrous ethanol was added to the cooled solution and maintained at 4 °C for 24 h. The precipitate was washed with anhydrous ethanol and lyophilized to obtain products with different degrees of selenylation (YP-LSe and YP-HSe).

### 2.3 Determination of structure and physicochemical properties

#### 2.3.1 Chemical composition analysis

The total polysaccharide content was determined using the phenol‑sulfuric acid method (Ru et al., 2020). The selenium content was determined using an inductively coupled plasma mass spectrometer (7800, Agilent, USA), as previously described for the ICP-MS method (Lin et al., 2022). Protein content was analyzed using the Coomassie brilliant blue method (Bradford, 1976). The *m*-hydroxybiphenyl method was used to determine the uronic acid content (Blumenkrantz & Asboe-Hansen, 1973). The sulfate content was measured by the turbidimetric method, with potassium sulfate as the standard (Dodgson & Price, 1962).

#### 2.3.2 Ultraviolet spectrum (UV) scan and Fourier transform-infrared (FT-IR) spectroscopy

The samples were scanned with UV-spectrophotometer (UV-2600, Shimadzu, Japan). The samples were mixed with KBr and pressed into thin slices, which were analyzed using an FT-IR (Nicolet iS50, Thermo Fisher, Germany) spectrometer in the frequency range of 4000 to 400 cm^−1^.

#### 2.3.3 Congo red analysis

The Congo red assay serves as a specialized method for identifying polysaccharides that exhibit a triple-helical conformation. Briefly, the sample solution was mixed with Congo red solution (0.23 mmol/L) in a volume ratio of 2:1, followed by the gradual addition of sodium hydroxide (NaOH) solution. A UV spectrophotometer was used to scan at 400–600 nm, and the λ max at different NaOH concentrations was recorded.

#### 2.3.4 Solubility and transmittance

The solubility of samples was determined according to the previously reported method (Feng, Qiu, et al., 2022). The sample (10 mg/mL) was centrifuged at 5000 r/min for 10 min, and the supernatant was dried to a constant weight and recorded. The transmittance was measured at 420 nm. Correspondingly, the solubility was calculated using Eq. (1).

(1) $$\mathrm{Solubility}\;\left(\%\right)=\frac{m_{1}}{m_{0}}\times 10$$

where m_0_ is the total sample mass, and m_1_ is the mass of the dried supernatant.

#### 2.3.5 Interface thickness

The interface thickness of samples was determined by previous methods (Lv et al., 2024). In brief, 10 mL of polysaccharide sample was mixed with 250 μL of polystyrene microsphere suspension and equilibrated for 2 h. The nanoparticle size of the mixture was determined by a nanoparticle size and Zeta-potential analyzer (NANO ZS90, Malvern, UK). The interface thickness was calculated using Eq. (2).

(2) $$d_{T}=\frac{d-d_{i}}{2}$$

where d_T_ represents the interfacial thickness of the polysaccharide samples, d represents the particle size of the polystyrene microspheres that have absorbed the sample, and d_i_ represents the particle size of the polystyrene microspheres, respecti*v*ely.

#### 2.3.6 Surface morphology observation by scanning electron microscope (SEM) and transmission electron microscope (TEM) analysis

The solid samples were fixed on a sample holder with conductive adhesive and sputtered with gold powder. The surface morphology of samples was observed by SEM (SU8010, Hitachi, Japan) and photographed at 500× and 1000× magnification. In addition, the morphology of polysaccharide samples also was observed by TEM (H-7650, Hitachi, Japan).

### 2.4 Functional properties

#### 2.4.1 Emulsifying properties

The emulsifying properties were assessed by turbidimetry (Ben Romdhane et al., 2017). The polysaccharide solutions (10 mg/mL) were mixed with soybean oil in a ratio of 3:1 (*v*/v). Subsequently, the emulsions were prepared by homogenizing the mixtures for 3 min using a high-speed mixer (T18, IKA, Germany) at 10,000 r/min. Aliquots of emulsion (0.1 mL) were taken at 0 and 10 min after homogenization and diluted with 10 mL of 1 mg/mL sodium dodecyl sulfate solution. A UV spectrophotometer was used to evaluate the absorbance at 500 nm. Eq. (3) and Eq. (4) were used to evaluate the emulsion activity index (EAI) and emulsion stability index (ESI), respectively.

(3) $$\mathrm{EAI}\;\left(\frac{m^{2}}{g}\right)=\frac{\left(2\times 2.303\times A_{0}\right)}{\varphi \times W\left(g\right)}$$

(4) $$\mathrm{ESI}\;\left(\%\right)=\frac{A_{10}}{A_{0}}\times 100$$

where A_0_ and A_10_ are the absorbance of the diluted emulsion for 0 min and 10 min, respectively. φ denotes oil volume fraction (0.25), and W (g) denotes polysaccharide mass.

#### 2.4.2 Foaming properties

The foaming properties were evaluated according to previously reported methods (Ben Romdhane et al., 2017). 12 mL of sample solution (10 mg/mL) was homogenized at 10,000 r/min for 2 min. The volume change of the sample solution was recorded at the end of homogenization and 30 min later. Eq. (5) and Eq. (6) were used to calculate foaming capacity (FC) and foaming stability (FS), respectively.

(5) $$\mathrm{FC}\;\left(\%\right)=\frac{V_{T}-V_{0}}{V_{0}}\times 100$$

(6) $$\mathrm{FS}\;\left(\%\right)=\frac{V_{30}-V_{0}}{V_{0}}\times 100$$

where V_0_, V_T_, and V_30_ represent the sample volume before homogenization, the volume after homogenization completion, and the volume after 30 min of standing, respectively.

#### 2.4.3 Antioxidant activity

The hydroxyl radical scavenging activity was determined after appropriate adjustment of the method reported by Zhao et al. (2023). Sample solutions (1.0–10.0 mg/mL), salicylic acid ethanol solution (4.5 mmol/L), and FeSO_4_ solution (4.5 mmol/L) were mixed in equal volumes. Subsequently, 6 mmol/L of H_2_O_2_ was added to the mixture, with a volume twice that of the sample. After reacting at 37 °C for 30 min, the absorbance was detected at 510 nm. The clearance rate was calculated according to Eq. (7).

(7) $$\mathrm{Hydroxyl radical scavenging rate}\;\left(\%\right)=\frac{A_{0}-\left(A_{X}-A_{X0}\right)}{A_{0}}\times 100$$

where, the blank group was replaced with deionized water and subjected to the same treatment, with absorbance expressed as A_0_. A_X_ represents the absorbance of the mixed system with sample. A_X0_ represents the absorbance of the mixed system without the chromogenic agent H_2_O_2_.

The scavenging activity against the DPPH radical of samples was evaluated with minor modifications of the method reported by Huang et al. (2022). Different concentrations of samples (1.0–10.0 mg/mL) were mixed with DPPH ethanol solution (0.2 mmol/L) at the same volume. After 30 min of reaction in the dark, the absorbance of the reaction products was detected at 517 nm. The clearance rate was calculated using Eq. (8).

(8) $$\mathrm{DPPH radical scavenging rate}\;\left(\%\right)=\frac{A_{0}-\left(A_{X}-A_{X0}\right)}{A_{0}}\times 100$$

where A_0_ represents the absorbance of the blank group. A_X_ is the absorbance of the mixed system with sample. A_X0_ represents the absorbance of the mixed system without DPPH.

In addition, the reducing power of polysaccharide samples was also evaluated (Zhao et al., 2023). Briefly, 0.5 mL of phosphate buffers (0.2 mol/L, pH 6.8) and 0.1 mL of 10 mg/mL K_3_Fe(CN)_6_ were added to 0.2 mL of sample solution (1.0–10.0 mg/mL). After incubation at 50 °C for 20 min, 0.5 mL of 0.1 g/mL trichloroacetic acid solution was added. The mixture was then centrifuged at 8000 r/min for 10 min. Subsequently, 0.5 mL of the supernatant was carefully withdrawn, after which 0.5 mL deionized water and 0.1 mL of 0.1 mg/mL FeCl_3_ solution were added sequentially. Finally, the absorbance of the resulting system was measured at 700 nm. The reducing power was determined using Eq. (9).

(9) $$A=A_{X}-A_{0}$$

A is the reducing power, A_X_ is the absorbance of the mixed system of samples, and A_0_ is the absorbance of the blank group.

### 2.5 *In vitro* digestion characteristics

#### 2.5.1 Simulated *in vitro* gastrointestinal digestion

The method reported by Wu et al. (2021) was used as a reference to prepare gastric electrolyte (SGE) juice and small intestinal electrolyte (SIE) juice. The sample solution (10 mg/mL) was mixed with an equal volume of SGE, ensuring that the final mixing system had a pepsin activity of 2000 U/mL. The pH was subsequently adjusted to 3.0, followed by incubation with the reaction mixture at 37 °C for 2 h. After mixing the gastric digestive solution with an equal volume of SIE, the trypsin activity in the final mixture reached 100 U/mL, with a bile salt concentration of 10 mmol/L. The pH was subsequently adjusted to 7.0, followed by incubation with the reaction mixture at 37 °C for 2 h. The gastric and gastrointestinal digestion samples were collected every 30 min for subsequent experiments. At the conclusion of each simulated digestion phase, the reaction was terminated by subjecting it to a boiling water bath for 5 min. Gastric and gastrointestinal digestion samples were centrifuged at 5000 r/min for 10 min, and the supernatant was retained for subsequent analysis. The gastric digestive products and gastrointestinal digestion products were named YP-G (YP-I), YP-LSe-G (YP-LSe-I), and YP-HSe-G (YP-HSe-I), respectively.

#### 2.5.2 Particle size and zeta potential

Particle size and Zeta potential of digested samples were determined using the nanoparticle size and Zeta potential analyzer.

#### 2.5.3 Total and reduced polysaccharides (CR)

CR content of samples during gastrointestinal digestion was determined by the 3,5-dinitrosalicylic acid (DNS) method (Feng, Bie, et al., 2022). The total polysaccharide content of the digested samples was analyzed using the method described in Section 2.3.1. Digestibility was calculated based on the initial CR, the total polysaccharide content at different stages, and the corresponding CR during *in vitro* digestion (Wu et al., 2021).

### 2.6 Statistical analysis

All experiments were performed in triplicate, and data were presented as mean ± standard deviation. The data were analyzed using one-way ANOVA with SPSS 26.0 software, followed by Duncan's Multiple Significant Difference Analysis to evaluate significant differences (*p* < 0.05).

## 3 Results and the discussion

### 3.1 Analysis of structure and physicochemical properties

#### 3.1.1 Chemical composition analysis

**Fig. 2:**
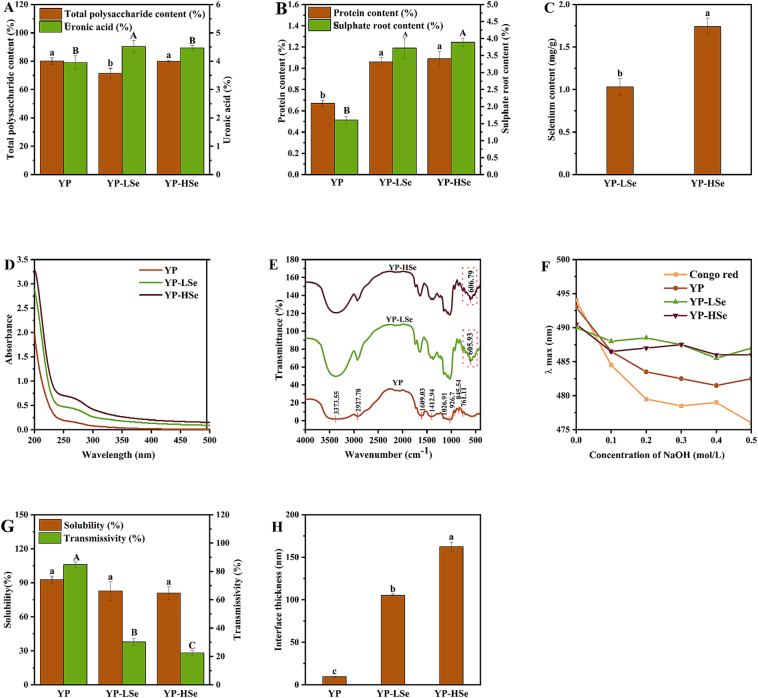
(A–C) The chemical composition of the sample. (D) The UV spectra of samples. (E) The FT-IR spectra of samples. (F) Maximum absorption λ_max_ of the Congo red–polysaccharide complex at various concentrations of NaOH. (G) Solubility and transmittance of samples. (H) Interfacial thickness of samples. (For interpretation of the references to colour in this figure legend, the reader is referred to the web version of this article.)

The results of the chemical composition of polysaccharide samples were presented in Fig. 2A–C. The total polysaccharide content of YP was 80.05 ± 2.47 %. The total polysaccharide content of YP-LSe and YP-HSe decreased to 71.05 ± 3.69 % and 79.86 ± 0.87 %, respectively. The total polysaccharide content of YP-HSe was significantly higher than that of YP-LSe, which suggested that there may be no direct relationship between the reduced total polysaccharide content and the extent of selenylation. During the selenylation process, the secondary alcohol precipitation step may lead to the loss of the total polysaccharide content. Similar to the present study, the total polysaccharide content of selenylated *Momordica charantia* L. polysaccharide decreased (Ru et al., 2020). Notably, the contents of uronic acids in YP-LSe and YP-HSe were 4.52 ± 0.22 % and 4.47 ± 0.08 %, respectively, while YP was 3.95 ± 0.25 %, which might be due to the slight degradation during the selenylation process (Feng, Qiu, et al., 2022). In addition, the protein and sulfate contents of the samples significantly increased after selenylation. However, there was no significant difference between YP-LSe and YP-HSe in terms of the content of uronic acids, proteins, and sulfates. The degree of selenylation in polysaccharides is intrinsically related to their functional properties; therefore, the selenium content serves as a pivotal parameter for characterizing the structural and functional attributes of selenylated polysaccharides. Due to the effective elimination of free selenium through ethanol washing during the sample preparation stage, the measured selenium was bound selenium attached to YP. The selenium content of YP-LSe and YP-HSe was 1.03 ± 0.10 mg/g and 1.74 ± 0.10 mg/g, respectively. This suggested that a portion of H_2_SeO_3_ molecules could effectively bind to YP, and increasing the amount of sodium selenite appropriately may help improve the degree of selenylation.

#### 3.1.2 UV and FT-IR analysis

The UV spectral data (Fig. 2D) revealed distinct absorption peaks for samples in the range of 260 to 280 nm, indicating the presence of a minor protein. Fig. 2E illustrated the absorption peaks of the samples within the FT-IR spectra. The intense and broad absorption peak observed at 3373 cm^−1^ and the distinct absorption peak at approximately 2927 cm^−1^ were attributed to the stretching vibrations of the O—H bond and the C—H bond, respectively (Deng et al., 2024). The absorption peaks located at approximately 1609 cm^−1^ and 1412 cm^−1^ were likely indicative of C=O stretching vibrations or the presence of COO^−^ groups (Wang et al., 2022). This suggested that the polysaccharide samples contained uronic acid. The peaks in the wavenumber range of 1000–1200 cm^−1^ indicated the presence of glycosidic bonds and pyranose rings. The obvious characteristic peak near 845 cm^−1^ was due to the tensile vibration of the α-glycosidic bond (Hong et al., 2021). In addition, YP-LSe and YP-HSe exhibited the same characteristic peaks as YP. Therefore, the YP extracted in this experiment was an acidic polysaccharide containing uronic acid and α-glycosidic bond. Moreover, compared to YP, the spectra of YP-LSe and YP-HSe revealed small peaks near 606 cm^−1^, which were attributed to the stretching vibration of Se—O—C. Similar bands were observed around the selenium-modified polysaccharides of *Hohenbuehelia serotina* (Wang et al., 2018). This confirmed the organic binding of the selenium-containing group to YP macromolecules during the selenylation process. The results of the FT-IR analysis were consistent with Fig. 2C, confirming that selenylation is an effective method for converting natural polysaccharides into selenium-containing polysaccharides.

#### 3.1.3 Congo red test

The triple helical structure of polysaccharides is integral to their functional properties. Glycosidic bonds and intramolecular hydrogen bonds contribute to the formation of the helical structure, while intermolecular hydrogen bonds and interactions between the polysaccharide and the solvent stabilize the triple helix structure (Meng et al., 2020). Trihelix polysaccharide possesses the ability to form complexes with Congo red molecules through its unique hydrophobic cavity, thereby inducing a change in the spatial conformation of Congo red, resulting in a significant λ max redshift (Guo et al., 2021). However, as depicted in Fig. 2F, the absence of a λ max redshift in the polysaccharide sample solutions suggested that Congo red did not effectively bind to the polysaccharide samples. This finding indicated a triple helical conformation was absent and suggested the possible existence of amorphous forms. The finding was in accordance with the outcomes of the Congo red test for selenylated dandelion polysaccharides (Wang et al., 2022). Optimal selenylation levels contributed to the formation of a highly ordered chain conformation, while excess selenium content may disrupt the inter and intramolecular forces, impairing the stability of the triple helical structure (Cheng et al., 2018).

#### 3.1.4 Solubility, transmittance, and interface thickness

The solubility of polysaccharide can reflect its chemical structure, chain conformation, and aggregates. As depicted in Fig. 2G, the solubility of YP was 92.77 ± 3.06 %, and there is no significant change during the selenylation process (*p* > 0.05). However, selenylation effectively increased the solubility of *Sagittaria sagittifolia* L. polysaccharides (Feng, Qiu, et al., 2022). The transmittance of YP, YP-LSe, and YP-HSe was 84.80 ± 1.91 %, 30.18 ± 2.54 %, and 22.48 ± 1.58 %, respectively. The transmittance of YP was higher than that of YP-LSe and YP-HSe. The transmittance of the samples decreased with the increase in selenylation, indicating that aggregates existed in YP-LSe and YP-HSe, and the degree of aggregation was positively correlated with selenium concentration.

The van der Waals force between droplets was reduced by their thicker interfacial layer, which correspondingly improved spatial stability and enhanced emulsion stability. Therefore, the interface thickness can be used to evaluate the steric hindrance changes in YP caused by selenylation, and the possibility of stabilizing emulsion of polysaccharide samples could be evaluated. As shown in Fig. 2H, the interface thickness of YP, YP-LSe, and YP-HSe were 9.51 ± 0.49 nm, 104.98 ± 1.81 nm, and 162.45 ± 5.59 nm, respectively. The interface thickness value increased with the increase in selenium content, which suggested that selenylation enhanced the steric hindrance effect of YP. Some studies showed that acetylation and other chemical modification treatments increased the thickness of the hydration layer on the surface of emulsified particles and enhanced the emulsifying properties (Huang et al., 2022).

#### 3.1.5 Surface morphology analysis

**Fig. 3:**
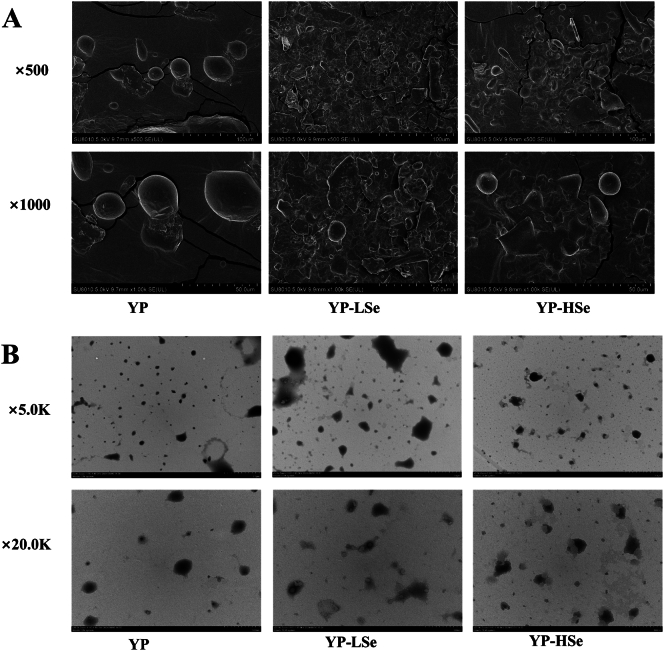
(A) SEM diagram of samples. (B) TEM diagram of samples.

The micromorphology of the polysaccharide samples was qualitatively observed by SEM (Fig. 3A). YP exhibited a near-spherical structure with a smooth surface. It was reported that the preliminarily extracted YP had a network structure of spherical or oval particles (Ma et al., 2017). The differences in the microscopic morphology of polysaccharides may be attributed to variations in their extraction methods, purification processes, and drying treatments. However, YP-LSe and YP-HSe predominantly exhibited lamellar and bladed structures, with spherical structures attached to rough surfaces. This was due to surface changes caused by intermolecular or van der Waals force interactions between selenium and polysaccharides during hygrothermal treatment in an acidic environment (Bo et al., 2021). The morphology of the samples under the TEM was shown in Fig. 3B. The TEM image of YP displayed a spherical structure, which was consistent with the results of SEM. The presence of selenium induced the aggregation of the polysaccharide samples.

### 3.2 Functional properties analysis

#### 3.2.1 Emulsifying properties

**Fig. 4:**
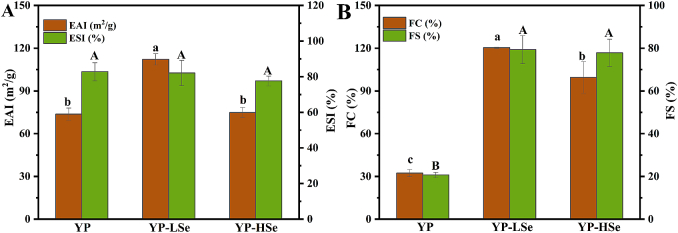
(A) Emulsification properties of samples. (B) Foaming properties of samples.

The emulsifying properties of selenylated polysaccharides were evaluated using the turbidity method (Fig. 4A). No significant difference in the ESI was detected among the YP and selenylated YP samples. However, the EAI values of the YP, YP-LSe, and YP-HSe were 73.70 ± 4.34 m^2^/g, 112.18 ± 3.96 m^2^/g, and 74.82 ± 3.61 m^2^/g, respectively. Selenylation increased the EAI of YP, but compared to YP-LSe, the EAI of YP-HSe decreased by 37.36 %, indicating that low selenylation significantly enhanced the emulsifying properties of YP. The bonding of selenium to the hydroxyl groups of the polysaccharide chain modified the balance between hydrophilic and hydrophobic moieties, thereby decreasing interfacial tension and enhancing emulsifying ability (Tang & Huang, 2022). However, excessive selenylation reduced the hydrophilicity of polysaccharide. The hydrogen bonding between polysaccharides diminished as the number of polysaccharide layers in the aqueous phase decreased (Yang et al., 2023). This reduction compromised the stability of the interfacial layer, ultimately leading to a decrease in emulsifying performance. In addition, the increase of uronic acid and protein content was closely related to the emulsifying activity of polysaccharide emulsion (Shen et al., 2024).

#### 3.2.2 Foaming properties

The FC and FS indices are used to quantify the capacity of the polysaccharide samples to adsorb and form stable membranes on the gas-water interface (Shen et al., 2024). Due to the presence of numerous hydroxyl groups, polysaccharides have hydrophilic characteristics, which help them to retain in the aqueous phase of the two-phase foam system. This characteristic helps to improve the stability and thickening properties of food products. As shown in Fig. 4B, the FC of YP, YP-LSe, and YP-HSe was 32.33 ± 2.52 %, 120.33 ± 0.47 %, and 99.50 ± 11.07 %, respectively. After selenylation, the FC of YP was significantly increased by 88.00 % and 67.17 %, respectively, which was consistent with the trend of EAI. A possible reason for this result might be that the viscosity of YP changed with the increasing selenium content. Additionally, the FS of YP, YP-LSe, and YP-HSe was 20.67 ± 1.21 %, 79.33 ± 6.60 %, and 77.83 ± 6.36 %, respectively. Compared with that of YP, the FS of YP-LSe and YP-HSe was significantly increased by 58.66 % and 57.16 %, indicating that the foam volume of YP-LSe and YP-HSe slightly decreased. Compared to YP-LSe, the FC and FS of YP-HSe were decreased, indicating that lower levels of selenylation were more conducive to the formation of an effective protective interface layer for YP.

#### 3.2.3 Antioxidant activity

**Fig. 5:**
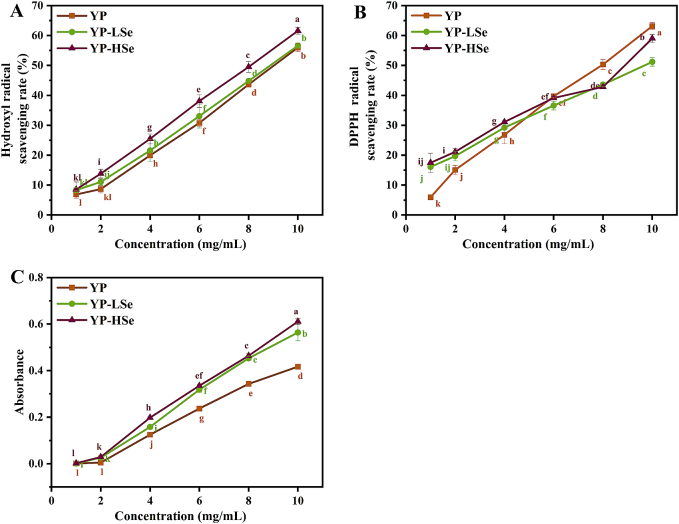
Antioxidant activity of samples. (A) Hydroxyl radicals scavenging rate. (B) DPPH radicals scavenging rate. (C) Reducing power.

As shown in Fig. 5A, the scavenging abilities of YP, YP-LSe, and YP-HSe against hydroxyl radicals increased with enhancing concentration within the tested range (1.0–10.0 mg/mL). The maximum scavenging rates of YP, YP-LSe, and YP-HSe were 55.98 ± 1.37 %, 56.62 ± 0.56 %, and 61.59 ± 1.18 %, respectively. There were significant statistical differences between different concentrations in the same sample, establishing a clear dose-response relationship. It is worth noting that at the same concentration, the scavenging capacity of YP against hydroxyl radicals was positively correlated with selenium content. Similarly, the scavenging activity of the DPPH radical was positively correlated with the sample concentration (Fig. 5B). The scavenging rates of YP-LSe and YP-HSe were significantly higher than YP within the concentration range of 1.0–4.0 mg/mL. However, at concentrations of 4.0–10.0 mg/mL, the scavenging rate of YP was higher than that of YP-LSe and YP-HSe. The increase in selenium modification rate was beneficial to the enhancement of antioxidant activity of polysaccharide (Wei et al., 2024). As shown in Fig. 5C, all YP samples had a marked increase in reducing power with increasing concentration. YP-HSe exhibited the highest reducing power at the same concentration, followed by YP-LSe and YP.

Numerous studies have shown that the antioxidant activity of polysaccharides is influenced by factors such as conformation, uronic acid content, senior structure, and molecular weight. Although it is currently unclear how these factors interact with each other to affect the antioxidant activity of polysaccharides, the following information can be inferred from the current study. All YP samples exhibited antioxidant capacity, including reducing power and hydroxyl radical scavenging capacity. The antioxidant activity of YP-HSe and YP-LSe was higher than that of YP, which was correlated with selenium content and high uronic acid content. Related studies have also highlighted the key role of selenium in selenium-containing polysaccharides on antioxidant activity *in vitro* (Cheng et al., 2018). The introduction of selenium-containing groups can activate the hydrogen atoms at the terminal carbon, and the ability of polysaccharides to provide hydrogen atoms increases with the enhanced activation degree of these groups (Wang, Ji, et al., 2024). Therefore, polysaccharides with higher selenium content exhibit stronger antioxidant activity compared to the original polysaccharides. The uronic acid content of polysaccharides has been reported as an important indicator of antioxidant activity. Negatively charged acidic polysaccharides can interact with metal ions to form ligand complexes, thereby scavenging free radicals. Additionally, based on the spatial and stereo-electronic effects of electron-withdrawing carboxyl groups, hydroxyl radicals selectively acquire hydrogen atoms from the C-5 of polysaccharides in the presence of uronic acid (Fernandes & Coimbra, 2023). The antioxidant activity of *Actinidia arguta* polysaccharide SPS3, which has the highest uronic acid content, was significantly greater than that of SPS2 and SPS1 with lower uronic acid content (Zhu, Zhang, et al., 2019). Additionally, spherical, lamellar, and honeycomb porous structures are known to enhance the antioxidant activity of polysaccharides (Chen, Jiang, et al., 2024). SEM observations in the present study showed that both YP-HSe and YP-LSe had a spherical lamellar structure, which may be responsible for the enhanced antioxidant activity.

### 3.3 *In vitro* digestive properties

#### 3.3.1 Changes in particle size and zeta potential during digestion

**Fig. 6:**
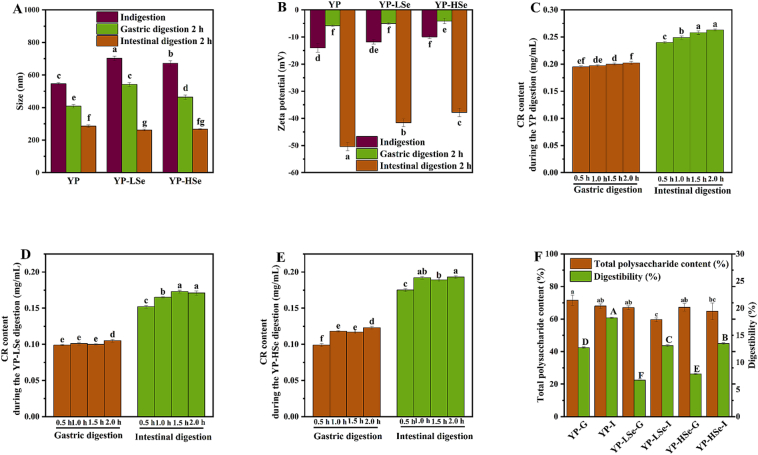
(A) Changes in particle size during digestion. (B) Changes in Zeta potential during digestion. (C–E) The CR of samples during digestion.

The changes in particle size and Zeta potential of the three polysaccharides during simulated digestion *in vitro* were shown in Fig. 6A&B. After gastric digestion, the particle size of YP, YP-LSe, and YP-HSe significantly decreased to 408.2 ± 11.0 nm, 541.7 ± 11.4 nm, and 463.7 ± 13.8 nm, respectively. Gastrointestinal digestion resulted in a further decrease in particle size of three samples, but there was no significant difference in particle size between YP-LSe-I and YP-HSe-I. The decrease in the particle size suggested that the combined action of pepsin, trypsin, and inorganic salts promoted polysaccharide hydrolysis. The Zeta potential of YP was −14.0 ± 1.60 mV, as YP was an acidic polysaccharide that exhibited a negative potential in solution, allowing it to acquire electrons. The absolute value of the Zeta potential of YP-LSe was slightly lower than YP, but significantly higher than YP-HSe, and the absolute value of the Zeta potential decreases with the increase of selenium content. Polysaccharides tend to aggregate in a solution, and the highly acidic gastric environment can enhance the intermolecular cohesion, thus destroying the system and reducing the stability. Consequently, the absolute values of the Zeta potential for the polysaccharide solutions experienced a significant reduction following gastric digestion. Upon completion of gastrointestinal digestion, the absolute values of the Zeta potential exhibited a marked increase, reaching 50.4 ± 1.5 mV, 41.6 ± 1.4 mV, and 37.8 ± 1.6 mV, respectively, indicating a more stable polysaccharide system. Such pronounced alterations in particle size and Zeta potential confirm the active participation of YP, YP-LSe, and YP-HSe in the *in vitro* simulated digestion process. Therefore, to further elucidate their digestive characteristics, CR, total polysaccharide content, and gastrointestinal digestibility were subsequently assessed during the digestion process.

#### 3.3.2 Changes of CR, total polysaccharide content, and digestibility during digestion

The assessment of CR content serves as a reliable indicator of polysaccharides degradation and the disruption of glycosidic bonds. The changes in CR content during the digestion of polysaccharide samples were presented in Fig. 6C–E. During simulated gastric fluid digestion, the CR values of YP, YP-LSe, and YP-HSe ranged from 0.195 to 0.202 mg/mL, 0.099 to 0.105 mg/mL, and 0.099 to 0.123 mg/mL, respectively. The highly acidic environment of the gastric fluid exerted an influence on the glycosidic bonds of polysaccharides, leading to degradation and the breaking of glycosidic bonds, which increased the number of reducing ends (Hu et al., 2013). During intestinal digestion, the CR content for YP samples was observed to range from 0.240 to 0.263 mg/mL, 0.152 to 0.173 mg/mL, and 0.175 to 0.193 mg/mL, respectively, suggesting a modest degradation of these polysaccharides throughout the gastrointestinal digestion process. This trend was analogous to the findings reported for *Artocarpus heterophyllus Lam*. polysaccharide during gastrointestinal digestion (Zhu, Yao, et al., 2019). Furthermore, these results were consistent with the observations for *Agaricus Bosporus* polysaccharide as described by Fu et al. (2023).

After gastrointestinal digestion, the total polysaccharide content of YP, YP-LSe, and YP-HSe was significantly decreased to 67.95 ± 1.43 %, 59.63 ± 1.13 %, and 64.73 ± 5.11 %, respectively (Fig. 6F). The digestibility of the three polysaccharides at various stages of digestion was also detailed in Fig. 6F. During gastric digestion, the digestibility of YP was 12.53 %, while in gastrointestinal digestion, its digestibility reached 17.28 %, indicating partial degradation of YP in the gastrointestinal environment. This finding was consistent with previous reports on okra (*Abelmoschus esculentus*) polysaccharides (Wu et al., 2021). The digestibility of YP-LSe and YP-HSe during gastric digestion was 6.75 % and 7.92 %, respectively, while those during gastrointestinal digestion were 12.40 % and 12.93 %, respectively, both lower than the corresponding values for YP. Owing to the persistent acidic environment created by selenylation, YP has undergone partial degradation. Consequently, the interaction between YP-LSe and YP-HSe and the enzymes during the digestion phase may be relatively restricted, thereby reducing further degradation of the samples and enhancing resistance in the gastrointestinal environment. It has been reported that polysaccharides with strong anti-digestibility are significantly utilized in the colon, playing key roles in regulating intestinal flora composition and maintaining energy homeostasis (Wu et al., 2021). The digestibility of YP-LSe was lower than that of YP-HSe, indicating that YP-LSe was more resistant to the gastrointestinal environment.

## 4 Conclusion

In this study, two types of selenylated yam polysaccharides (YP-LSe and YP-HSe) were successfully obtained by selenium of yam polysaccharides in the HNO_3_-Na_2_SeO_3_ selenite system. Both YP-LSe and YP-HSe preserved the conformation of polysaccharides and displayed the structural features of the polysaccharide chains; in addition, selenylated samples simultaneously exhibited more diverse structural forms. The single spherical structure of YP exhibited layered and foliar structures after selenylation. Moreover, there were slight changes in the chemical compositions of YP-LSe and YP-HSe, with a significant increase in selenium content. The higher steric hindrance effect of YP-LSe and YP-Hse increased their interfacial thicknesses and improved their emulsifying and foaming properties. Compared with YP, YP-LSe and YP-HSe exhibited higher antioxidant activities, such as hydroxyl radical scavenging capacity and reducing power, with YP-HSe showing significantly higher activity than YP-LSe. This may be related to the synergistic effect of YP and selenium content, microstructure, glyoxylate content, and other factors. YP samples were partially degraded under gastrointestinal digestion conditions, but YP-LSe and YP-HSe exhibited stronger gastrointestinal tolerance. This study provides a theoretical basis for the utilization of selenylated YP as selenium-rich dietary supplements and antioxidants. In future research, we will focus on studying the effect of polysaccharides on the bioavailability of selenium after digestion and conduct *in vivo* experiments to comprehensively evaluate the structure-activity relationship between polysaccharides and selenium.

## CRediT authorship contribution statement

**Weiling Liu:** Writing – original draft, Investigation. **Yujun Jiang:** Supervision, Software. **Jia Shi:** Writing – review & editing, Supervision.

## Declaration of competing interest

The authors declare that they have no known competing financial interests or personal relationships that could have appeared to influence the work reported in this paper.

## Data Availability

Data will be made available on request.
